# A Feedback Regulatory Loop Containing McdR and WhiB2 Controls Cell Division and DNA Repair in Mycobacteria

**DOI:** 10.1128/mbio.03343-21

**Published:** 2022-03-31

**Authors:** Wei Zhou, Shaojia Huang, Bridgette M. Cumming, Yong Zhang, Wei Tang, Adrie J. C. Steyn, Shiyun Chen, Yangbo Hu

**Affiliations:** a CAS Key Laboratory of Special Pathogens and Biosafety, Wuhan Institute of Virology, Center for Biosafety Mega-Science, Chinese Academy of Sciences, Wuhan, China; b University of Chinese Academy of Sciences, Beijing, China; c Africa Health Research Institute, University of KwaZulu Natal, Durban, South Africa; d Department of Microbiology, University of Alabama at Birmingham, Birmingham, Alabama, USA; e State Key Laboratory of Virology, Wuhan Institute of Virology, Center for Biosafety Mega-Science, Chinese Academy of Sciences, Wuhan, China; Washington University School of Medicine in St. Louis

**Keywords:** *Mycobacterium tuberculosis*, stress response, drug resistance, transcriptional regulation, Wbl, Rv1830

## Abstract

Cell division must be coordinated with DNA repair, which is strictly regulated in response to different drugs and environmental stresses in bacteria. However, the mechanisms by which mycobacteria orchestrate these two processes remain largely uncharacterized. Here, we report a regulatory loop between two essential mycobacterial regulators, McdR (Rv1830) and WhiB2, in coordinating the processes of cell division and DNA repair. McdR inhibits cell division-associated *whiB2* expression by binding to the AATnACAnnnnTGTnATT motif in the promoter region. Furthermore, McdR overexpression simultaneously activates *imuAB* and *dnaE2* expression to promote error-prone DNA repair, which facilitates genetic adaptation to stress conditions. Through a feedback mechanism, WhiB2 activates *mcdR* expression by binding to the cGACACGc motif in the promoter region. Importantly, analyses of mutations in clinical Mycobacterium tuberculosis strains indicate that disruption of this McdR-WhiB2 feedback regulatory loop influences expression of both cell growth- and DNA repair-associated genes, which further supports the contribution of McdR-WhiB2 regulatory loop in regulating mycobacterial cell growth and drug resistance. This highly conserved feedback regulatory loop provides fresh insight into the link between mycobacterial cell growth control and stress responses.

## INTRODUCTION

Mycobacterium tuberculosis, one of the most successful bacterial pathogens, is a major threat to global health (1). The difficulty in eradicating M. tuberculosis is related to its complex transcriptional regulatory network controlling cell growth and survival under different stress conditions (2, 3). Cell division is one of the most important physiological processes for bacterial growth and must be coordinated with DNA replication and repair to maintain the ploidy of offspring (4–6). However, the regulation of these two fundamentally important processes in M. tuberculosis is largely unknown.

M. tuberculosis encodes approximately 200 transcriptional regulators (TRs) to control bacterial cell growth and, thus, cope with environmental stress responses. Previous characterizations of transcriptional regulatory networks using chromatin immunoprecipitation sequencing (ChIP-seq) have identified the targets of most TRs in M. tuberculosis (7, 8). However, the physiological roles of these regulatory target pairs have not been characterized. Subsaturation levels of mutagenesis with a random transposon or CRISPR interference screening have determined that a quarter of total genes are essential for growth in M. tuberculosis, including several TRs (9–14). This suggests that these TRs participate in the regulation of essential mycobacterial growth processes.

MerR family proteins are widely known to increase adaptability in different bacterial species (15). These proteins regulate gene expression by binding with repeat sequences in promoter regions and normally contain three domains: the N-terminal DNA-binding domain, the C-terminal effector binding domain, and the linker region (15). Most MerR proteins respond to metal ions, antibiotics, or drug-like compounds and activate the transcription of detoxification-related genes to eliminate the toxicity of substances (15, 16). However, some MerR proteins, such as HonC and GlnR (17, 18), inhibit the expression of their target genes, indicating an alternative regulatory mechanism of MerR proteins. Rv1830 is a MerR family protein that has been characterized as an essential regulator in M. tuberculosis (9, 10, 19), but its regulatory role has not been characterized. Recently, a whole-genome sequence comparison of clinically isolated M. tuberculosis strains suggested a role for Rv1830 in drug resistance (20). However, the link between the roles of Rv1830 in drug resistance and in essential growth processes is not clear.

The WhiB-like (Wbl) family of proteins, which contain four invariant cysteine residues that form an O_2_- and NO-sensitive [4Fe-4S] cluster, are unique to actinomycetes and play versatile regulatory roles in virulence (21) and antibiotic resistance (22, 23) in M. tuberculosis. WhiB2 is an essential transcriptional regulator involved in the regulation of cell division (24). Knockdown or overexpression of *whiB2* resulted in the formation of filamentous cells (24–26). Furthermore, the expression of *whiB2* was decreased during M. tuberculosis infection in mice (27), and M. tuberculosis cells showed a filamentous shape in macrophages (28). These results suggest that the expression of WhiB2 is regulated in the process of M. tuberculosis infection.

In this study, we report that Rv1830 regulates mycobacterial cell division and survival under stress conditions; thus, we rename this protein McdR (mycobacterial cell division regulator). We show that McdR differentially regulates the expression of the cell division-associated gene *whiB2* and the DNA repair-associated genes *imuAB* and *dnaE2*. Moreover, we demonstrate that WhiB2 regulates the expression of *mcdR* to form a highly conserved feedback regulatory loop. Our study provides incentive to investigate other feedback regulatory loops enabling mycobacterial cell growth in the presence of stress.

## RESULTS

### McdR regulates mycobacterial growth and participates in stress responses.

**FIG 1:**
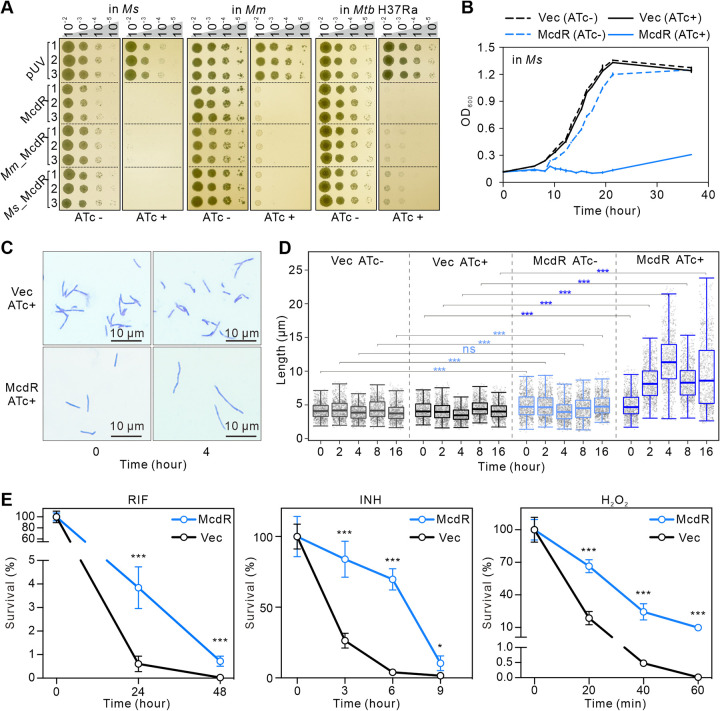
McdR regulates mycobacterial cell division and participates in stress responses. (A and B) Overexpression of *mcdR* inhibits mycobacterial cell growth on 7H10 plates (A) or in 7H9 broth (B). The concentrations of ATc were 50 ng/mL for M. smegmatis or 200 ng/mL for M. tuberculosis H37Ra and M. marinum. Error bars indicate the SD (standard deviations) from three independent experiments. (C) Microscopic observation of M. smegmatis when *mcdR* was overexpressed (McdR, ATc+) compared with the vector control (Vec, ATc+) at the indicated times. (D) Cell length of M. smegmatis when *mcdR* was overexpressed compared with the vector control at the indicated times. In the boxplots, the 25th and the 75th percentiles are boxed, and the lengths of individual bacteria (1,000 cells) are shown as gray dots. Thick lines indicate the mean values in each group. (E) Survival rate of M. smegmatis when *mcdR* was overexpressed compared with the vector control under RIF, INH, and H_2_O_2_ treatments, as calculated by CFU counting. Error bars indicate the SD from three independent experiments.

Sequence alignments showed that McdR was conserved in both slow- and fast-growing mycobacteria (see Fig. S1 in the supplemental material), and the identity of McdR proteins among M. tuberculosis, Mycobacterium smegmatis (*MSMEG_3644*), and Mycobacterium marinum (*MMAR_2707*) was greater than 75%. We first attempted to delete the M. smegmatis *mcdR* homologue named *MSMEG_3644* but could not obtain any mutant clones. However, this gene could be deleted when M. tuberculosis *mcdR* was expressed on an integrating plasmid in M. smegmatis (Fig. S2). These data suggest that *mcdR* is an essential gene in M. smegmatis, which is consistent with previous transposon screening in M. tuberculosis demonstrating essentiality (9, 10, 19). Therefore, we overexpressed M. tuberculosis *mcdR*, M. marinum *mcdR*, or M. smegmatis *mcdR* in M. tuberculosis, M. marinum, or M. smegmatis and found that they all efficiently inhibited mycobacterial cell growth (Fig. 1A and B). Morphological analysis showed that cells of McdR-overexpressing strains were filamentous and longer than those of the vector control at different growth stages (Fig. 1C and D and Fig. S3A and S3B). Together, these data suggest that overexpression of McdR inhibits mycobacterial cell division.

10.1128/mbio.03343-21.1 FIG S1 Multiple-sequence alignment of McdR protein sequences from different mycobacterial species. Identical residues are highlighted in red, and amino acids with greater than 50% similarity are boxed in a blue frame. Download FIG S1, TIF file, 2.7 MB. Copyright © 2022 Zhou et al. 2022 Zhou et al. https://creativecommons.org/licenses/by/4.0/ This content is distributed under the terms of the Creative Commons Attribution 4.0 International license.

10.1128/mbio.03343-21.2 FIG S2 Confirmation of *MSMEG_3644* deletion when *mcdR* from M. tuberculosis was expressed in M. smegmatis. (A) Schematic diagram of *MSMEG_3644* deletion in M. smegmatis. Dotted frame represents the deleted region. (B) PCR confirmation of *MSMEG_3644* deletion in M. smegmatis. Primers used are indicated in panel A. Download FIG S2, TIF file, 2.5 MB. Copyright © 2022 Zhou et al. 2022 Zhou et al. https://creativecommons.org/licenses/by/4.0/ This content is distributed under the terms of the Creative Commons Attribution 4.0 International license.

10.1128/mbio.03343-21.3 FIG S3 McdR regulates M. smegmatis cell division and participates in stress responses. (A and B) SEM images (A) and statistical analysis (B) of M. smegmatis with (McdR) or without (Vec) McdR overexpression. (C to E) Survival rates of M. smegmatis with (*mcdR*-kd) or without (vec) *mcdR* knockdown under RIF, INH, and H_2_O_2_ treatments, as calculated by CFU counting. Error bars indicate the SD from three independent experiments. Download FIG S3, TIF file, 2.7 MB. Copyright © 2022 Zhou et al. 2022 Zhou et al. https://creativecommons.org/licenses/by/4.0/ This content is distributed under the terms of the Creative Commons Attribution 4.0 International license.

Considering the close relationship between the filamentous phenotype and stress responses (28, 29), we compared the survival rates of M. smegmatis with or without *mcdR* overexpression in the presence of different stresses, i.e., isoniazid (INH; 60 μg/mL), rifampicin (RIF; 30 μg/mL), or hydrogen peroxide (H_2_O_2_; 5 mM). As shown in Fig. 1E, overexpression of *mcdR* in M. smegmatis significantly increased cell survival under each of these stresses. Consistent with this, the *mcdR* knockdown strain showed increased cell sensitivity to INH, RIF and H_2_O_2_ (Fig. S3C to E). Together, our data suggest that McdR regulates mycobacterial cell division and susceptibility to anti-TB drugs and oxidative stress.

### McdR acts as a cell cycle checkpoint regulator.

**FIG 2:**
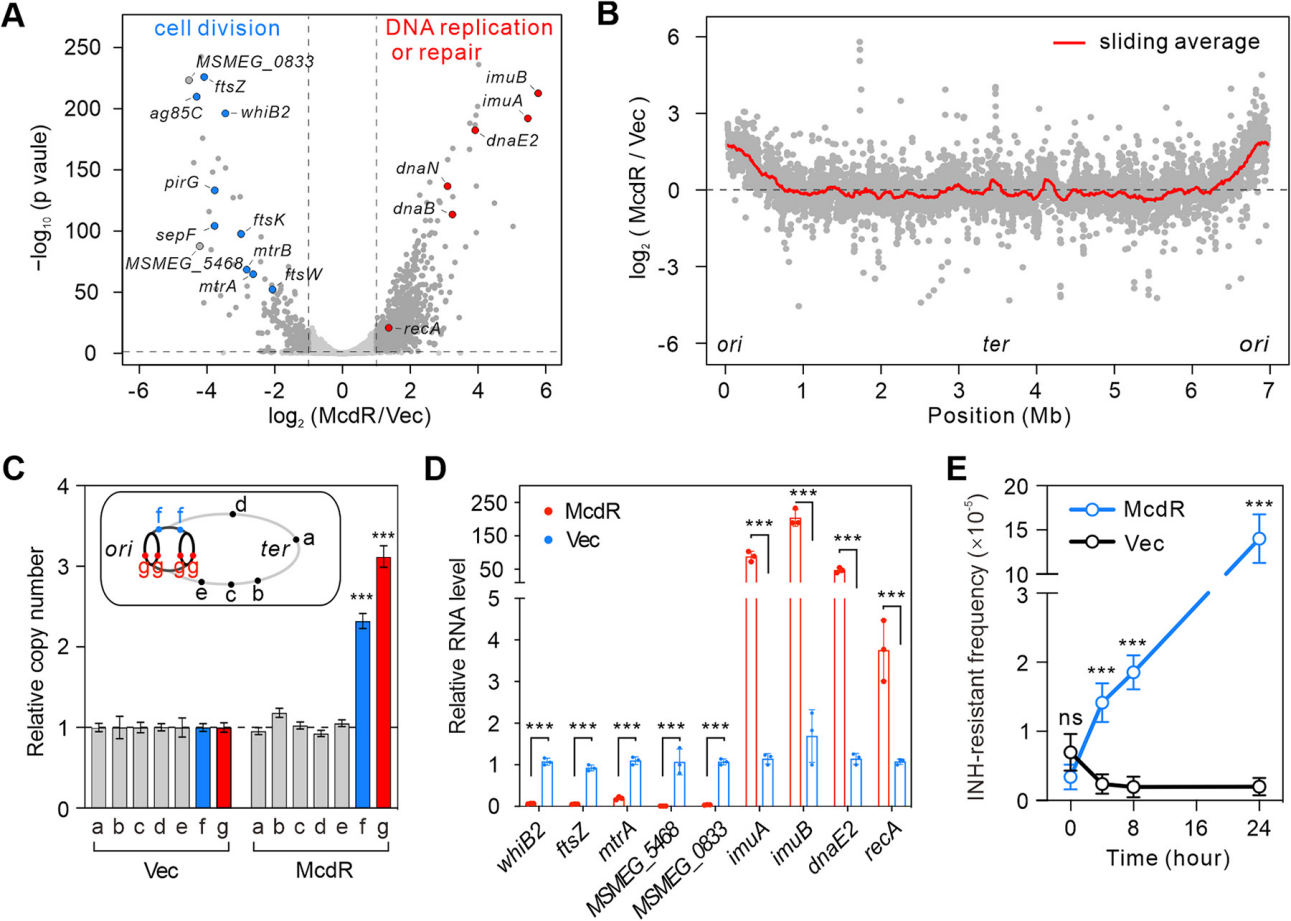
Overexpression of McdR inhibits cell division and promotes DNA repair in mycobacteria. (A) Gene expression changes observed between overexpression and normal expression of *mcdR* in M. smegmatis. Genes associated with cell division are indicated with blue dots, while those related to DNA replication and repair are indicated with red dots. (B) Log_2_ fold changes in the genome-wide mRNA levels (depicted by dark gray dots for each gene) and a 150-gene sliding window average (depicted by the red line) of M. smegmatis with or without *mcdR* overexpression. (C) Relative copy numbers of genomic DNA located at differential regions in M. smegmatis with or without *mcdR* overexpression. The locations of fragments a to g in the M. smegmatis chromosome are indicated. The DNA copy number was tested by qPCR assay. The mean and SD were calculated from three independent measurements. (D) Relative mRNA levels of *whiB2*, *ftsZ*, *mtrA*, *MSMEG_5468*, *MSMEG_0833*, *imuAB*, *dnaE2*, and *recA* in M. smegmatis with or without *mcdR* overexpression. Bars and error bars show the means and SD calculated from three independent qRT-PCR measurements. (E) The mutation frequency of M. smegmatis with (McdR) or without (Vec) *mcdR* overexpression induced by treatment with 15 μg/mL INH. Mean and SD calculated from three measurements are shown.

To further investigate what is regulated by McdR at the global level, we employed RNA-seq to compare the gene expression profiles of M. smegmatis with or without *mcdR* overexpression. As shown in the volcano plot in Fig. 2A and Table S2, overexpression of *mcdR* repressed cell division-associated genes, including *whiB2*, *mtrAB*, *ag85C*, *sepF*, *pirG*, and several *dcw* (division and cell wall) genes, like *ftsKWZ* (30) (indicated with blue color), which is consistent with the inhibitory effects of *mcdR* overexpression on mycobacterial cell division and the filamentous phenotype (Fig. 1A to D). The *mcdR* overexpression also activated genes involved in DNA replication and repair, including *imuAB*, *dnaE2*, *recA, dnaB and dnaN* (indicated with red color). In addition, genes located closer to the origin of replication (*ori*) generally showed higher mRNA levels in the *mcdR*-overexpressing strain (Fig. 2B), suggesting that *mcdR* overexpression increases the DNA copy numbers near the *ori*. In support of our hypothesis, genes closer to the *ori* had higher copy numbers than those distal from the *ori* when *mcdR* was overexpressed, as tested by quantitative PCR (qPCR) assay (Fig. 2C).

10.1128/mbio.03343-21.8 TABLE S2 RNA-seq data for M. smegmatis with or without McdR overexpression. Download Table S2, XLSX file, 0.5 MB. Copyright © 2022 Zhou et al. 2022 Zhou et al. https://creativecommons.org/licenses/by/4.0/ This content is distributed under the terms of the Creative Commons Attribution 4.0 International license.

We next confirmed the regulatory roles of McdR with genes associated with cell division as well as DNA replication and repair by quantative reverse transcription-PCR (qRT-PCR) (Fig. 2D). As previous studies have demonstrated roles for *imuAB* and *dnaE2* in mutagenesis and in *in vivo* survival (31–33), we next calculated mutation frequency of M. smegmatis strains with or without *mcdR* overexpression. As shown in Fig. 2E, overexpression of *mcdR* increased the mutation frequency by 70-fold for INH resistance. Given the roles of McdR in the repression of cell division and activation of DNA replication or repair, we propose that McdR functions as a cell cycle checkpoint regulatory protein.

### McdR regulates *whiB2* expression by binding to an AATnACAnnnnTGTnATT motif.

**FIG 3:**
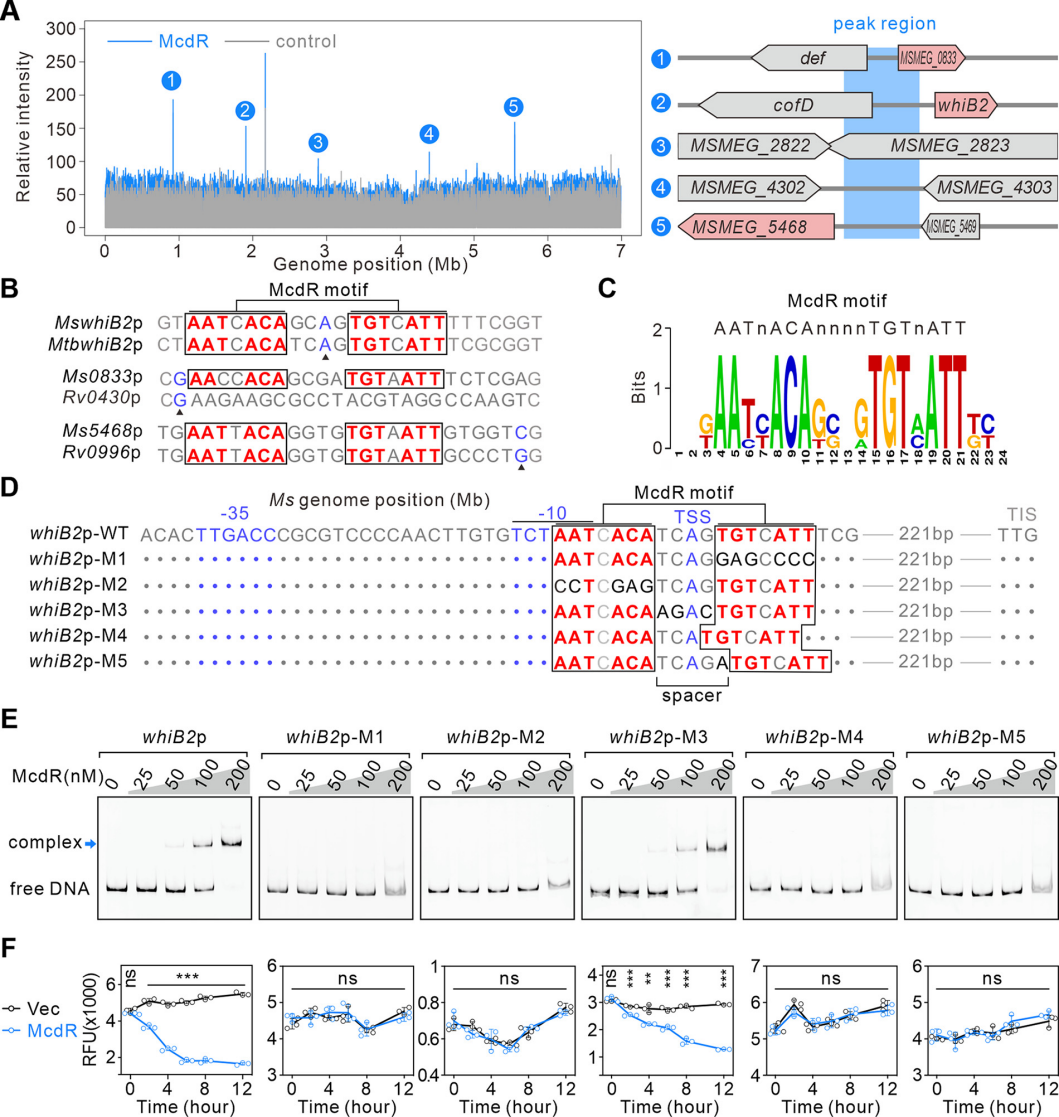
McdR directly inhibits the expression of *whiB2*. (A) Relative counts of sequencing reads mapped to the M. smegmatis genome excluding ribosome RNA regions in the DIP-Seq assay for groups using McdR with strep-tag (McdR) or without strep-tag (control). The detailed locations of the five peaks specifically enriched in the McdR group are indicated on the right. (B) Analysis of McdR motif in promoters of *whiB2*, *MSMEG_0833*, *MSMEG_5468* genes in M. smegmatis and their homologues in M. tuberculosis. Triangles indicate TSSs identified in M. tuberculosis. (C) The weblogo of McdR motif based on sequences shown in panel B. (D) Wild-type and mutated promoter sequences of the *whiB2* gene in M. tuberculosis used for EMSA. The proposed −35 element, −10 element, transcription start site (TSS), and translation initiation site (TIS) are indicated. The nucleotides consistent with *whiB2*p-WT are simplified as dots. McdR-binding sites are boxed. (E) Interaction between McdR and *whiB2* promoters analyzed by EMSA. (F) Comparison of *whiB2* promoter activities in M. smegmatis with or without overexpression of McdR. Data shown are the mean RFU and SD calculated from three independent measurements.

To further investigate the molecular regulatory mechanism of McdR, we performed a DNA immunoprecipitation sequencing (DIP-seq) assay to characterize the direct targets of McdR. Our results showed that McdR directly binds to the upstream regions of the *whiB2*, *MSMEG_0833*, and *MSMEG_5468* genes (Fig. 3A and Table S3), whose expression was inhibited by McdR overexpression (Fig. 2A and D). These data suggest the direct regulation of these targets by McdR. We next analyzed conserved sequences in promoters of these genes (including their homologs in M. tuberculosis) using multiple-sequence alignment and generated a potential McdR motif as AATnACAnnnnTGTnATT (Fig. 3B and C). We next screened the McdR motif in the promoter regions of M. tuberculosis and M. smegmatis and found that, in addition to these three targets, this motif also exists in several other genes (Table S4), suggesting a broad regulatory role of McdR in mycobacteria.

10.1128/mbio.03343-21.9 TABLE S3 Richness of McdR bound fragments in M. smegmatis mc^2^155 strain (NC_008596.1). Download Table S3, XLSX file, 0.01 MB. Copyright © 2022 Zhou et al. 2022 Zhou et al. https://creativecommons.org/licenses/by/4.0/ This content is distributed under the terms of the Creative Commons Attribution 4.0 International license.

Since WhiB2 has been shown to regulate bacterial cell division (24–26), we next focused on characterizing the regulatory relationship between McdR and WhiB2. The potential McdR motif AATnACAnnnnTGTnATT is located around the previously identified transcription start site (TSS) (34); therefore, we constructed mutations to test the role of this potential McdR motif in the regulation of McdR on the *whiB2* promoter (*whiB2*p) (Fig. 3D). McdR directly binds to the wild-type *whiB2*p to inhibit *whiB2* expression (Fig. 3E and F), but this regulatory effect was abolished when the reverse complementary sequence in AATnACAnnnnTGTnATT was mutated (*whiB2*p-M1 and *whiB2*p-M2) (Fig. 3D to F) or the spacer length was changed (*whiB2*p-M4 and *whiB2*p-M5) (Fig. 3D to F). However, mutating the spacer sequence without changing the spacer length had no effect (*whiB2*p-M3) (Fig. 3D to F).

To further confirm the connection between McdR and the AATnACAnnnnTGTnATT sequence, we performed electrophoretic mobility shift assay (EMSA) to test the binding of McdR with other promoters containing the AATnACAnnnnTGTnATT motif (Fig. S4A). McdR successfully binds with the promoter regions of *MSMEG_0833* (*Ms0083*p), *MSMEG_5468* (*Ms5468*p), and *Rv0996* (*Rv0996*p, homologous of *MSMEG_5468*) but not with the *Rv0340* promoter (*Rv0430*p, homologous of *MSMEG_0083*) (Fig. S4A), as only *Rv0430*p did not contain the AATnACAnnnnTGTnATT motif (Fig. 3B). DNA mutations of the McdR motif in *Ms0083*p abolished McdR binding (Fig. S4B). These data further indicated that McdR directly binds to the AATnACAnnnnTGTnATT motif to regulate the expression of its target genes. Both McdR protein and the McdR motif AATnACAnnnnTGTnATT in *whiB2*p are conserved (Fig. S5A), suggesting that the regulation of McdR to *whiB2*p would be widely applied in mycobacteria.

10.1128/mbio.03343-21.4 FIG S4 McdR recognizes the AATnACAnnnnTGTnTGT motif to directly regulate its targets. (A) The interaction of McdR and its targets in M. smegmatis or the homologous genes of the targets in M. tuberculosis. (B) The interaction of McdR and *Ms0833*p or mutated promoters. Download FIG S4, TIF file, 2.9 MB. Copyright © 2022 Zhou et al. 2022 Zhou et al. https://creativecommons.org/licenses/by/4.0/ This content is distributed under the terms of the Creative Commons Attribution 4.0 International license.

10.1128/mbio.03343-21.5 FIG S5 Neighbor-joining phylogenetic tree of McdR-*whiB2*p (A) and WhiB2-*mcdR*p (B) sequences in mycobacteria. The numbers at nodes represent bootstrap values. The M. tuberculosis complexes are labeled red. Corynebacterium glutamicum was used as an outgroup. Download FIG S5, TIF file, 2.9 MB. Copyright © 2022 Zhou et al. 2022 Zhou et al. https://creativecommons.org/licenses/by/4.0/ This content is distributed under the terms of the Creative Commons Attribution 4.0 International license.

### WhiB2 feedback regulates *mcdR* expression by recognizing the cGACACGc motif.

**FIG 4:**
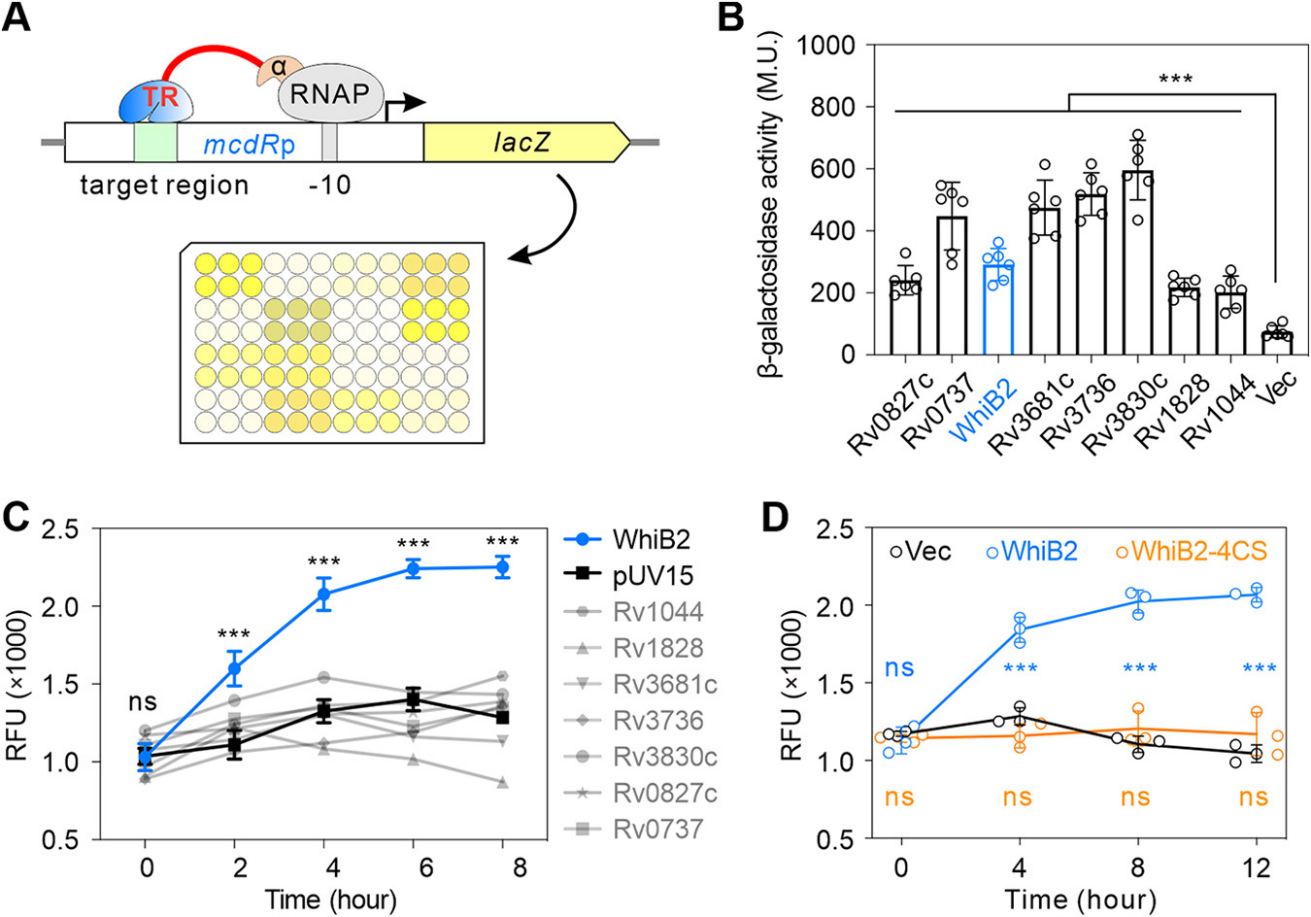
WhiB2 activated the expression of *mcdR*. (A) Diagram showing the bacterial one-hybrid system. RNAP, RNA polymerase; α, RNA polymerase subunit alpha; TR, transcriptional regulator. (B and C) The effects of different regulatory proteins on *mcdR* promoter activity in E. coli using bacterial one-hybrid system (B) or in M. smegmatis using a promoter-mCherry reporter system (C). M.U. represents Miller unit. (D) *mcdR* promoter activities in M. smegmatis when WhiB2 or WhiB2-4CS was overexpressed in M. smegmatis. Means and SD from three independent measurements are shown.

As *mcdR* is an essential gene in mycobacteria and its regulatory target, *whiB2*, is also stringently regulated (24, 26), we used a bacterial one-hybrid system (35) to screen the regulatory effects of transcriptional regulatory proteins on the *mcdR* promoter in E. coli (Fig. 4A). Eight transcriptional regulatory proteins were found to regulate the expression of *mcdR* in this assay (Fig. 4B). Among them, overexpression of *whiB2* successfully activated the expression of *mcdR* in M. smegmatis using an mCherry reporter system (Fig. 4C). Consistent with previous data showing that transcriptional regulation of Wbl family proteins depends on their conserved cysteine residues (36), activation of WhiB2-mediated *mcdR* expression was abolished when the four conserved cysteine residues were mutated to serine (WhiB2-4CS) (Fig. 4D).

**FIG 5:**
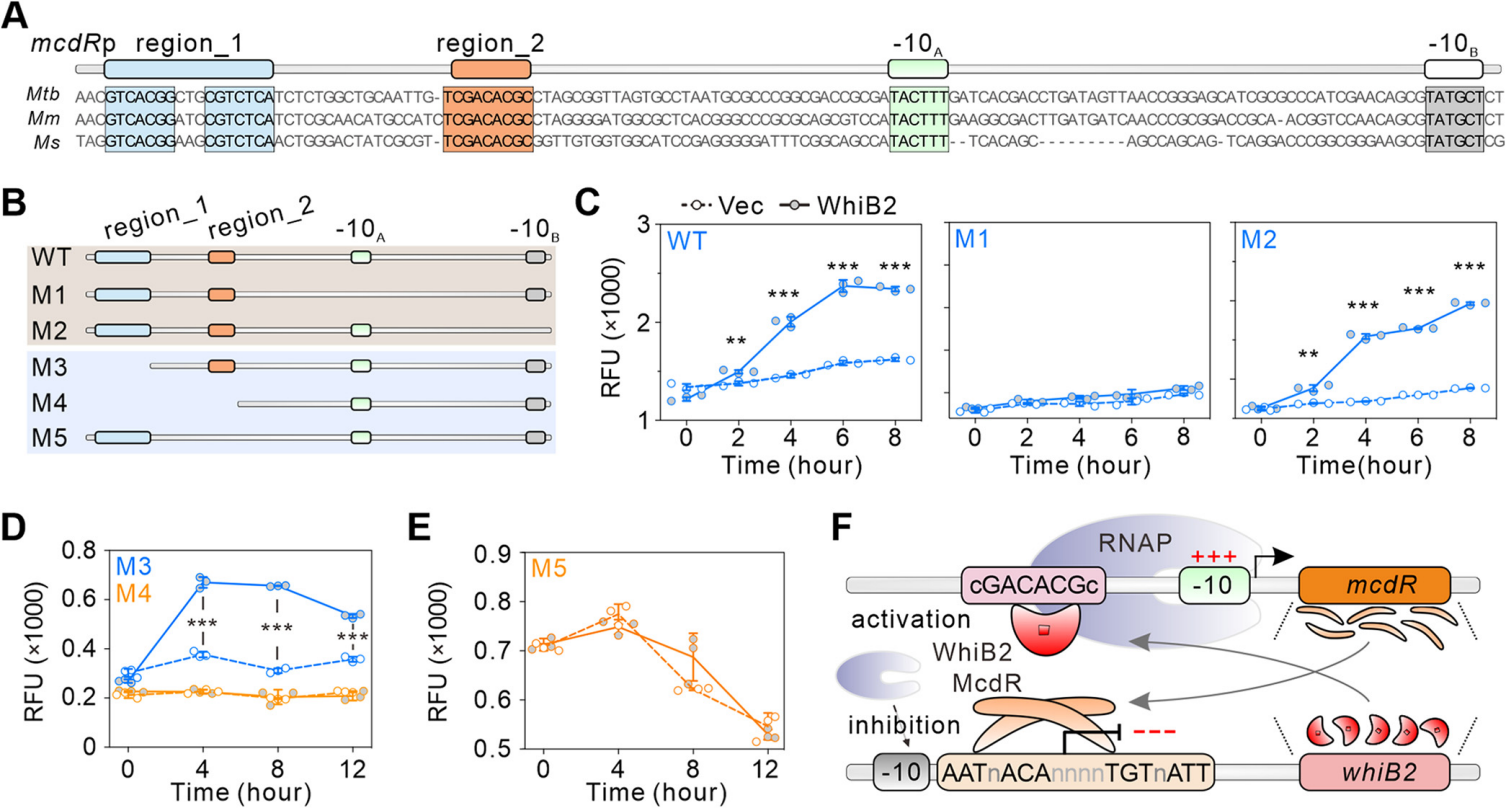
WhiB2 activates *mcdR* expression by recognizing the cGACACGc motif. (A) Promoter sequence alignments of the *mcdR* gene in M. smegmatis, M. marinum, and M. tuberculosis. Two conserved regions (region_1 and region_2) and two proposed −10 elements (−10_A_ and −10_B_) are indicated. (B) Diagram showing mutations in the *mcdR* promoter. (C to E) Relative promoter activities of *mcdR* wild type (C) and different mutations (M1 to M5) in M. smegmatis when WhiB2 was overexpressed compared with the vector control. Mean RFU and SD calculated from three independent measurements are shown. (F) A proposed feedback regulation loop containing McdR and WhiB2.

Further multiple-sequence alignment of the promoter sequences of *mcdR* and its homologous genes in M. marinum and M. smegmatis identified two conserved regions (region_1 and region_2) upstream of the two characterized TSSs (34) (Fig. 5A). We named the two −10 elements upstream of each TSS −10_A_ and −10_B_. Mutation of −10_B_ (M2), but not −10_A_ (M1), was activated by WhiB2 (Fig. 5B and C), indicating that WhiB2 activation is dependent on −10_A_ in the *mcdR* promoter. Deletion of region_1 (M3) had no effect on the activation, but deletion of both region_1 and region_2 (M4) abolished this activation (Fig. 5B and D). Furthermore, WhiB2 did not activate the promoter containing a mutation in region_2 (M5) (Fig. 5E). These data suggest that the WhiB2-mediated regulation of *mcdR* promoter (*mcdRp*) is facilitated by the region_2 sequence TCGACACGC. In addition, the phylogeny of WhiB2 and the promoter sequence of *mcdR* in mycobacterial species suggest that the WhiB2-mediated regulation of *mcdR*p is conserved (Fig. S5B).

To obtain the overall targets regulated by WhiB2, we searched the promoters of M. smegmatis and M. tuberculosis H37Rv (Table S4) for the characterized binding sequence TCGACACGC. We identified several potential targets, including *plcA*, *clpX*, and *Rv1405c*. Sequence alignments identified a putative WhiB2-binding motif as cGACACGc (Fig. S6A). In agreement with this finding, the promoter activities of M. tuberculosis *plcA*, *clpX*, and *Rv1405c* were activated by the overexpression of *whiB2* but not by the mutated allele coding for WhiB2-4CS (Fig. S6B).

10.1128/mbio.03343-21.6 FIG S6 WhiB2 activates the expression of its targets by binding to the cGACACGc motif. (A) The promoter sequences of *mcdR*, *plcA*, *clpX*, and *Rv1405c*. The weblogo of *mcdR*, *plcA*, *clpX*, and *Rv1405c* promoters is shown in the lower panel. (B) The promoter activities of the potential targets of WhiB2 (*plcA*, *clpX*, and *Rv1405c*) with overexpression of WhiB2 or WhiB2-4CS in M. smegmatis. Mean RFU and SD from three independent measurements are shown. Download FIG S6, TIF file, 2.8 MB. Copyright © 2022 Zhou et al. 2022 Zhou et al. https://creativecommons.org/licenses/by/4.0/ This content is distributed under the terms of the Creative Commons Attribution 4.0 International license.

10.1128/mbio.03343-21.10 TABLE S4 WhiB2 and McdR binding region in M. tuberculosis and M. smegmatis genome. Download Table S4, XLSX file, 0.04 MB. Copyright © 2022 Zhou et al. 2022 Zhou et al. https://creativecommons.org/licenses/by/4.0/ This content is distributed under the terms of the Creative Commons Attribution 4.0 International license.

Together, we conclude that WhiB2 binds to the cGACACGc sequence in the *mcdR* promoter to activate the expression of *mcdR*. In turn, McdR binds to the AATnACAnnnnTGTnATT motif in the *whiB2* promoter to inhibit the expression of *whiB2*. This feedback regulatory loop is important for precise regulation of mycobacterial cell division (Fig. 5F).

### Single nucleotide polymorphisms of *mcdR* influence its regulatory effect.

**FIG 6:**
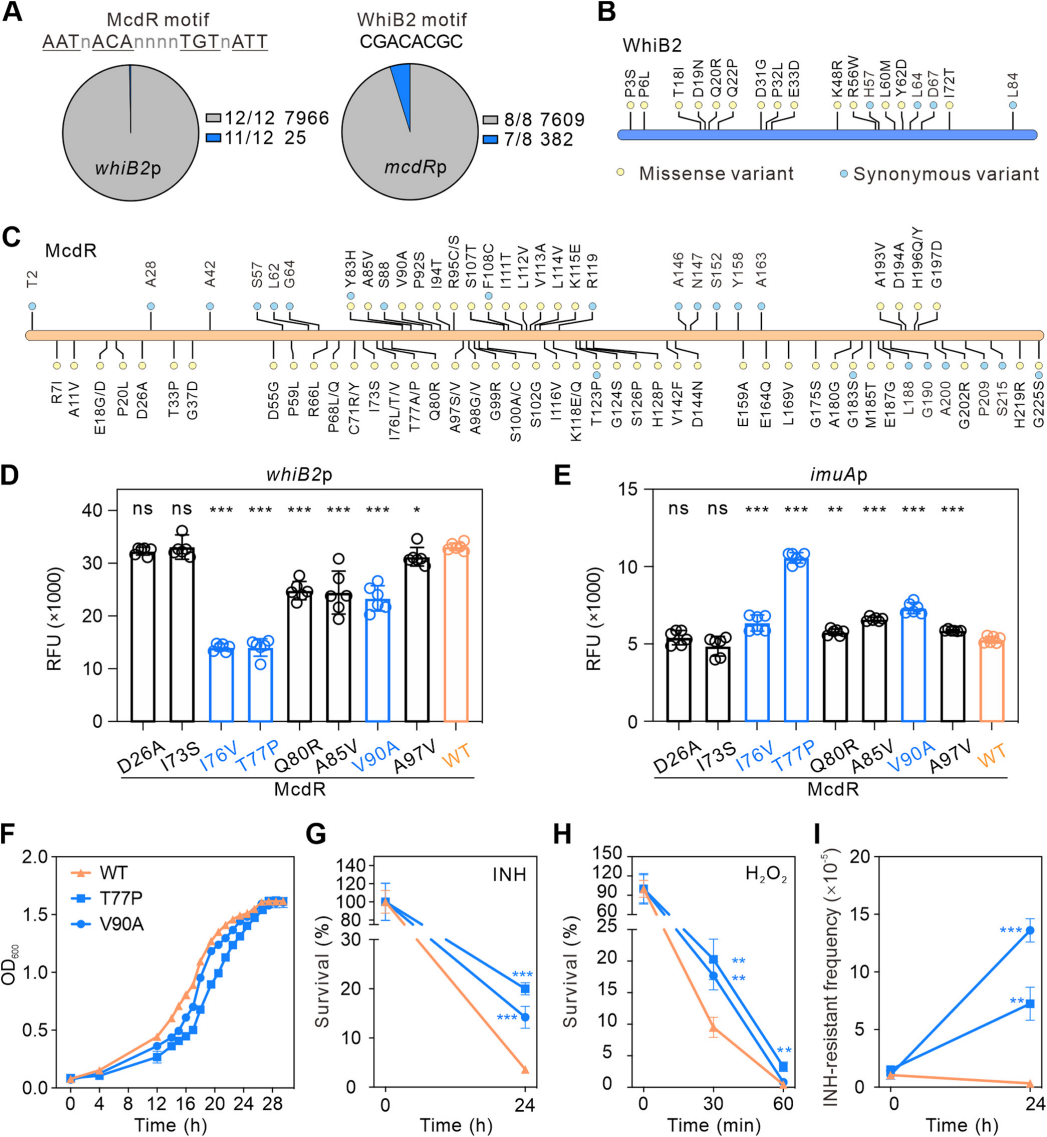
SNPs of *mcdR* influence the mycobacterial growth and stress responses. (A) The conserved binding sites of McdR and WhiB2 in the *whiB2* (left) and *mcdR* (right) promoters. Numbers of strains with different conservation in nucleotides are listed. (B and C) Distribution of SNPs in WhiB2 (B) and McdR (C) proteins in M. tuberculosis clinical strains. (D and E) Relative expression of *whiB2*p (D) and *imuA*p (E) in M. smegmatis containing different SNPs of the *mcdR* gene. (F) Growth curves of M. smegmatis containing different SNPs of the *mcdR* gene. (G and H) Survival rate of M. smegmatis containing different SNPs of the *mcdR* gene compared with the strain containing wild-type *mcdR* gene under INH (G) and H_2_O_2_ (H) treatments. (I) Mutation frequency of M. smegmatis strains containing wild-type (WT) or different SNPs of *mcdR* gene. Error bars indicate the SD from three tests.

Since both McdR and WhiB2 are essential regulators and the feedback loop regulates the fundamental process of cell division and participates in stress responses, we posited whether this feedback regulation had been disturbed in some M. tuberculosis clinically isolated strains. Hence, we analyzed the coding sequences and promoter regions of *mcdR* and *whiB2* in 7,991 sequenced clinical M. tuberculosis strains in the NCBI database. We found that the McdR and WhiB2 binding sites are conserved in each other’s promoters (Fig. 6A). However, the coding sequences (CDS) of McdR and WhiB2 contain several SNPs, some of which lead to changes in amino acid sequences (Fig. 6B and C). Subsequently, we tested whether the expression of *whiB2* and the *imuAB* operon would be influenced by SNPs in McdR. We replaced the *mcdR* gene in M. smegmatis with M. tuberculosis *mcdR* carrying different SNPs using an integrated plasmid. As is evident in Fig. 6D and E, D26A and I73S in McdR had no significant effect on the expression of *whiB2* and the *imuAB* operon. However, WhiB2 expression was inhibited by SNPs I76V, T77P, Q80R, A85V, V90A, or A97V, and the expression of *imuAB* operon was increased in these strains. Consistent with this, the growth of strains with SNP T77P or V90A showed slower growth (Fig. 6F) and increased survival rate in the presence of INH or H_2_O_2_ (Fig. 6G and H) compared with the strain containing the wild-type *mcdR* gene. Moreover, these two SNPs also increased the mutation rate for INH resistance (Fig. 6I). These data confirmed that disruption of McdR regulatory influences on mycobacterial stress responses.

## DISCUSSION

In this study, we show that McdR forms a feedback regulatory loop with WhiB2 to control mycobacterial cell division. Overexpression of McdR activates the expression of *recABCD*, *imuAB*, and *dnaE2* to increase DNA mutagenesis. Collectively, our results demonstrate that McdR may function as a cell cycle checkpoint regulator in mycobacteria to coordinate cell division and DNA repair during unfavorable environmental conditions.

Cell division is a key physiological process in bacteria that must be carefully coordinated with DNA replication or repair and is strictly regulated (37). Through transcriptome sequencing (RNA-seq) and phenotype analyses, we showed that McdR directly regulates the expression of *whiB2* (Fig. 2 and 3), which is a known essential regulator of mycobacterial cell division (24–26). Furthermore, we showed that WhiB2 regulates the expression of *mcdR* through a feedback mechanism by binding to the cGACACGc motif located upstream of the −10 promoter element (Fig. 5), which is consistent with the recently characterized model proposed for the mode of action of the WhiB family of proteins (38, 39). Interestingly, the cGACACGc motif was also found in the promoters of several other genes, including *ftsBHW*, *plcA*, *clpX*, and *Rv1405c* (Table S4, Fig. S6), and their expression was repressed when WhiB2 was inactivated by *mcdR* overexpression (Table S2). Several *dcw* genes were repressed upon McdR overexpression (Table S2). In addition to the WhiB2 binding site identified upstream of *ftsBHW*, we also identified one potential McdR binding site in the *wag31* promoter (Table S4), suggesting McdR also directly regulates the *dcw* genes in controlling mycobacterial growth. The MtrA/B complex is known to regulate several genes, including, but not limited to, *dacB1*, *sepF*, *fbpB*, *ripA*, and *ftsI*, which are associated with mycobacterial growth (40–42). The expression of *mtrAB* together with their targets was also inhibited by McdR overexpression (Table S2), but no McdR binding site was observed in the *mtrAB* promoter. Therefore, we hypothesize that McdR indirectly interacts with the MtrA/B regulatory network and directly regulates the expression of WhiB2 and the *dcw* genes to control cell division.

Causing DNA damage is a common mechanism by which antibiotics kill bacteria (43–45). However, DnaE2 is an error-prone DNA polymerase involved in DNA repair but lacks proofreading activity, which results in more mutations being introduced during DNA repair (31, 32, 46). In M. tuberculosis, DnaE2 increases mutagenesis and directly promotes the emergence of drug resistance, which plays a vital role in *in vivo* survival (31). ImuA/B are essential accessory factors for DnaE2, as they interact with DnaE2 and are required for mutagenesis in M. tuberculosis (32). Our data showed that overexpression of McdR activates the expression of *imuAB* and *dnaE2* and increases the DNA mutation rate for INH resistance (Fig. 2E). Consistent with our data, a previous study analyzed the whole-genome sequences of 594 clinical M. tuberculosis strains and found that mutations in the *mcdR* gene are associated with drug resistance (20). We propose that McdR acts as a bifunctional transcriptional regulator by inhibiting mycobacterial division and concurrently activating DNA repair mediated by *imuAB* and *dnaE2*.

M. tuberculosis can undergo dormancy in a nonreplicating state, causing latent infection (47), in which state the bacteria were highly tolerant to antibiotics and stresses (48, 49). It has been reported that the regulation of *whiB2*, *ftsKWZ*, *pbpB*, and *ripA* is important for filamentous cell formation, which promotes the development of mycobacterial dormant cells (50). Our data show that overexpression of *mcdR* effectively inhibits the expression of these genes, which may increase the tolerance of mycobacteria to stressful environments. In the meantime, the activation of *imuAB* and *dnaE2* upon McdR overexpression may also protect mycobacteria against DNA damage under stressful conditions. Together, those data suggest a role of McdR in controlling the formation of dormant cells and stress responses. Although most of our studies were performed in M. smegmatis, sequence alignments show that McdR (Fig. S1) and WhiB2 (51) are highly conserved in M. tuberculosis and M. smegmatis, and their binding sites are also conserved in most of their target promoters (Table S4). Therefore, we hypothesize that our proposed regulation model of McdR and WhiB2 in this study also work in M. tuberculosis, although further studies are required to confirm it.

MerR family regulators are known to bind with a reverse complementary sequence located in 19- or 20-bp spacer regions between promoter −35 and −10 elements, which in turn bends the promoter region for RNA polymerase recognition and activates the expression of targeted genes (52, 53). However, our results showed that McdR binds with the reverse complementary sequence AATnACAnnnnTGTnATT around the TSS but not in the promoter spacer region (Fig. 3), suggesting that McdR acts in an way analogous to that of the nonclassical MerR family protein HonC (17). These different characteristics imply that McdR acts uniquely to regulate the transcription of its target genes. In this study, we characterized the repressive effects of McdR on the *whiB2* promoter, but whether and how McdR directly activates its targets requires further study.

In summary, we have revealed a previously uncharacterized feedback regulatory loop mediated by two essential genes in mycobacteria. This conserved regulatory loop not only plays a vital role in the coordination of cell division and DNA repair but also participates in drug resistance and stress responses in mycobacteria. Our results provide fundamental insight into uncovering the link between mycobacterial cell growth control and stress responses.

## MATERIALS AND METHODS

### Bacterial strains and growth conditions.

The bacterial strains used in this study are summarized in Table S1 in the supplemental material. Escherichia coli strains were cultured in Luria-Bertani (LB) broth or on LB agar-solidified plates at 37°C. Mycobacterial cells were grown in 7H9 (Difco) liquid medium supplemented with 0.2% (wt/vol) glucose, 0.015 M NaCl, 0.2% (vol/vol) glycerol, and 0.05% (vol/vol) Tween 80 or on 7H10 (Difco) agar plates supplemented with 0.5% (vol/vol) glycerol at 37°C. For M. tuberculosis and M. marinum, 10% oleic acid-albumin-dextrose-catalase (Difco) was added.

10.1128/mbio.03343-21.7 TABLE S1 The strains, plasmids, and primers used in this study. Download Table S1, XLSX file, 0.02 MB. Copyright © 2022 Zhou et al. 2022 Zhou et al. https://creativecommons.org/licenses/by/4.0/ This content is distributed under the terms of the Creative Commons Attribution 4.0 International license.

### Plasmid constructions.

The plasmids and oligonucleotides used in this study are listed in Table S1. To construct recombinant plasmids, the target fragments and linearized vectors were amplified by PCR and cloned using a ClonExpress II one-step cloning kit (Vazyme, China). Mutations in the genes or promoters cloned in plasmids were introduced by following the protocol provided by the QuikChange II XL site-directed mutagenesis kit (Stratagene).

### Mutant construction and complementation.

M. smegmatis mutants were constructed as previously described (54). Briefly, pMV306-Hyg-McdR-Rv expressing wild-type or mutated McdR protein was transformed into M. smegmatis to form an McdR-overexpressing strain named *Ms*-*mcdR*. A pNILRB4 plasmid (kanamycin resistance) (55) carrying two fragments upstream and downstream of the *mcdR* (*MSMEG_3644*) gene then was transformed into *Ms*-*mcdR*. The single-crossed strains were selected by plating on 7H10 agar plates containing kanamycin. The double-crossed strains were selected by plating on 7H10 agar plates with 10% sucrose.

### Protein purification.

The McdR protein was expressed in E. coli BL21(DE3) with a C-terminal His tag or with both His tag and Twin-Strep tag using pET21a-McdR or pET21a-McdR-SH plasmid, respectively, and was purified as described previously (54). Briefly, bacterial cell pellets were collected and lysed by ultrasonication. The supernatant was collected and the proteins were first purified using a 5-mL HisTrap HP column (GE Healthcare). The elution fractions were collected and further purified using a Heparin column (GE Healthcare) and Superdex 200 Increase 10/300 GL column (GE Healthcare).

### DNA-binding analysis.

Electrophoretic mobility shift assays (EMSAs) were performed as described previously (16), with minor modifications. Briefly, around 200-bp fluorescein-labeled promoter fragments were amplified by PCR and extracted by a gel extraction kit (Omega). Promoter fragments (30 nM) were incubated with McdR in TB buffer (20 mM Tris-HCl, pH 7.9, 50 mM NaCl, 5 mM MgSO_4_, 1 mM dithiothreitol, 0.1 mM EDTA, 5% glycerol) at 37°C for 15 min. Samples were then loaded on 6% native 0.5× TBE-PAGE gel and run at 100 V. Gels were scanned by an Amersham Typhoon scanner (GE Healthcare).

### Promoter activity analysis in mycobacteria.

The promoter activity analysis in mycobacteria was performed as described previously (54). Mycobacterial promoters were fused to a promoterless *mCherry* gene in the pMV306 plasmid (56) and then cotransformed with the McdR overexpression plasmid based on pUV15TetORm (57) into M. smegmatis. The expression of McdR was induced by adding 50 ng/mL anhydrotetracycline (ATc) at an optical density at 600 nm (OD_600_) of ≈0.4. The promoter activities were indicated by relative fluorescence units (RFU; fluorescence intensities per unit of OD_600_) as detected by Bio-TEK Synergy H1. Assays were performed in duplicate in three independent experiments.

### Detection of genomic DNA copy numbers.

To detect copy numbers of DNA fragments located in different genomic regions, genomic DNA was extracted from M. smegmatis cells with or without McdR overexpression (50 ng/mL ATc for 2 h) using a TIANamp bacterial DNA kit (Tiangen, China). The copy numbers of seven different positions in the M. smegmatis genome were measured by qPCR, which was performed using iTaq universal SYBR green supermix (Bio-Rad) with 10 ng genomic DNA. The locations of seven positions in M. smegmatis genome (NC_008596) are the following: a, 2827073 to 2827178; b, 4304581 to 4304830; c, 5129805 to 5129950; d, 5336288 to 5336486; e, 1906450 to 1906563; f, 7476 to 7661; g, 6986398 to 6986515. Primers used for detection of fragments a to g are summarized in Table S1.

### RNA extraction, qRT-PCR, and RNA-seq analyses.

RNA extraction was performed as described previously (54, 58), with modifications. Cells with or without McdR overexpression (50 ng/mL ATc for 2 h) were harvested and ground in liquid nitrogen. RNA was extracted using TRIzol (Invitrogen) by following the manufacturer’s protocol. qRT-PCR was performed as previously described (54) using iTaq universal SYBR green supermix (Bio-Rad). The expression level of the *sigA* gene was used as an internal control. The qRT-PCR data were analyzed by CFX Manager (Bio-Rad). For RNA-seq experiments, rRNA was removed by a Ribo-off rRNA depletion kit (Vazyme). RNA libraries were constructed by using the NEBNext Ultra directional RNA library prep kit for Illumina (NEB). Sequencing was performed on the Illumina HiSeq X 10 platform using 2 × 150-bp paired-end sequencing. FastQC (59) and Trim Galore were used to trim the raw data. Reads were mapped to M. smegmatis genome (NC_008596) using BWA (60) and SAMtools (61). The gene expression levels were analyzed by DESeq2 (62) in R package (version 3.2.2), and genes were considered differentially expressed at fold change of ≥2 and adjusted *P* value of <0.05.

### DIP-seq analyses.

DIP-seq was performed as described previously (63), with modifications. The M. smegmatis genomic DNA was sheared into fragments with a peak at 250 bp by ultrasonication (Covaris M220). McdR (with or without Twin-Strep at the C terminus, 4 μM) and sheared DNA (4 μM) were incubated in TB buffer at 37°C for 20 min and cross-linked using 1% formaldehyde. Magnetic beads (Strep-Tactin XT; IBA) were added to select the McdR-DNA complex. DNA libraries were constructed by the NEBNext Ultra II FS DNA library prep kit (NEB). Sequencing was performed on the Illumina HiSeq X 10 platform using 2 × 150 bp paired-end sequencing. The analyses of sequencing reads were similar to those of RNA-seq. The relative intensity was calculated using reads counts of test groups (McdR with Twin-Strep tag) related to those of control groups (McdR without Twin-Strep tag).

### Microscopic observation.

Cell pellets were collected and resuspended in phosphate-buffered saline (PBS). Bacterial smears were applied on microscope slides, stained with crystal violet (1%), and observed with an optical microscope (Olympus BX53F). Cell length of M. smegmatis was measured by cellSens (Olympus). For scanning electron microscopy (SEM) observation, mycobacterial cells overexpressing McdR for 2 h were collected and washed 10 times with PBS. Cells were fixed with glutaraldehyde (2.5%), washed with PBS, and dehydrated again. Samples were then air dried, coated with gold, and scanned by SEM (Hitachi SU8010). M. smegmatis cell length was measured by ImageJ (64).

### Bacterial one-hybrid assay.

The *mcdR* promoter was fused to the promoterless *lacZ* gene in the pZT100 plasmid (65) and transformed into the E. coli K-12 Δ*lacZ* strain to obtain a reporter strain named K-12 *mcdR*p-*lacZ*. The coding regions of 178 transcriptional regulators were fused to the *rpoA* gene in the pOVR200 plasmid (58) and then transformed into the K-12 *mcdR*p-*lacZ* strain. The strains were cultured to an OD of ≈0.8 to test β-galactosidase activity as described previously (58). The data were calculated from three clones in duplicate.

### Detection of survival rate and mutagenesis rate.

To detect the survival rate under different stress conditions, M. smegmatis cells were cultured to an OD_600_ of ≈0.4 and diluted into 7H9 medium to a concentration of approximately 10^7^ CFU. Rifampicin (RIF), isoniazid (INH), or hydrogen peroxide (H_2_O_2_) was added to final concentrations of 30 μg/mL, 60 μg/mL, and 5 mM, respectively. The number of CFU was determined at different time points. To detect the mutation frequency, the number of CFU of M. smegmatis strains with or without *mcdR* overexpression was determined by spreading on 7H10 plates or 7H10 plates containing INH (15 μg/mL) at the indicated time points. The mutation frequency was calculated as number of CFU with INH divided by number of CFU without INH.

### M. smegmatis *mcdR* knockdown strain construction.

The M. smegmatis *mcdR* knockdown strain was constructed using a CRISPRi system as described previously (66). Briefly, plasmids pTetInt-dCas9 and pGrna2-*MsmcdR* (targeting M. smegmatis *mcdR* gene) were cotransformed into M. smegmatis. Colonies on plates with kanamycin (25 μg/mL) and hygromycin B (50 μg/mL) were selected and inoculated in 7H9 medium to a concentration of approximately 10^7^ CFU. ATc (50 ng/mL) was used to induce the expression of dCas9 and single guide RNA for 6 h to knock down the *MSMEG_3644* expression, and then RIF, INH, or H_2_O_2_ was added to final concentrations of 30 μg/mL, 60 μg/mL, and 5 mM, respectively. The number of CFU was determined at indicated time points.

### Phylogenetic tree and SNP analyses.

To construct the phylogenetic tree, the amino acid sequences of McdR and WhiB2 from different strains were downloaded from NCBI and aligned using ClustalW (67). Their neighbor-joining trees were created with MEGAX (68). The promoter sequences of the *mcdR* and *whiB2* genes were also aligned using ClustalW. For single nucleotide polymorphism (SNP) analysis, the sequencing reads were downloaded from the NCBI Sequence Read Archive (SRA). FastQC and Trim Galore were used to quality control raw data. Reads were mapped to the M. tuberculosis H37Rv genome (NC_000962) using BWA and SAMtools. BCFtools (69) was applied for SNP calling.

### Statistical analysis.

The raw data or mean values and standard errors (SD) are shown in each figure. The *P* values shown were calculated using two-tailed Student's *t* test: not significant (ns), *P* > 0.05; *, *P* ≤ 0.05; **, *P* ≤ 0.01; ***, *P* ≤ 0.001.

### Data availability.

The data set generated during this study is available upon reasonable request. RNA-seq and DIP-seq data reads had been submitted to the NCBI Sequence Read Archive (SRA) under accession numbers PRJNA760667 and PRJNA760668.
