# Supplementary figures and images for: A Feedback Regulatory Loop Containing McdR and WhiB2 Controls Cell Division and DNA Repair in Mycobacteria
Source: mBio. 2022 Mar 31;13(2):e03343-21. doi: 10.1128/mbio.03343-21 (PMC9040748; doi:10.1128/mbio.03343-21)

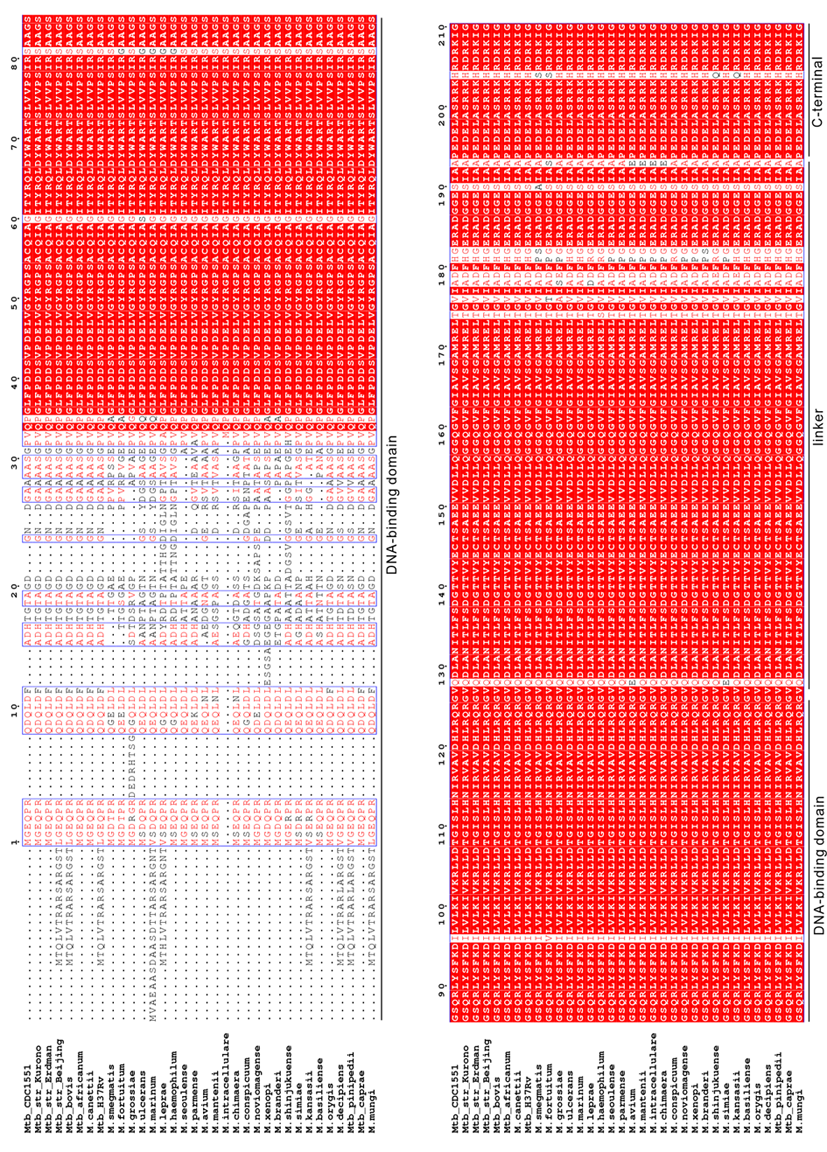

Supplement: FIG S1 [file mbio.03343-21-sf001.tif]

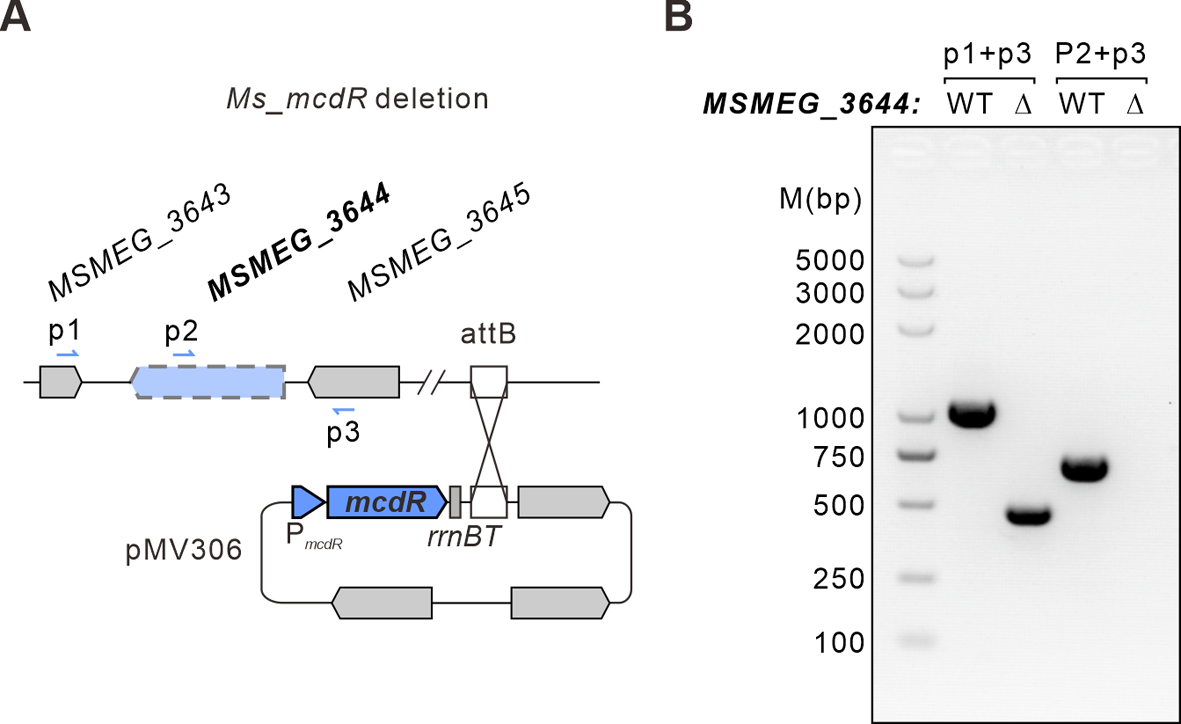

Supplement: FIG S2 [file mbio.03343-21-sf002.tif]

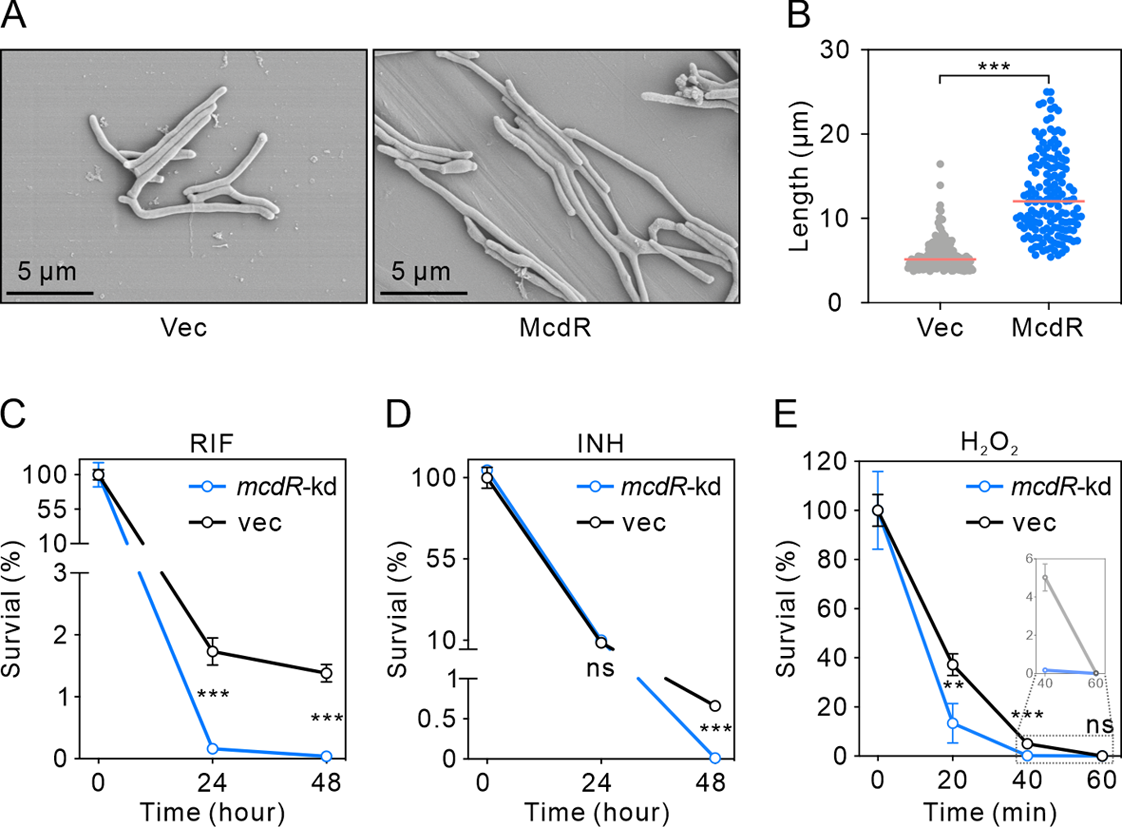

Supplement: FIG S3 [file mbio.03343-21-sf003.tif]

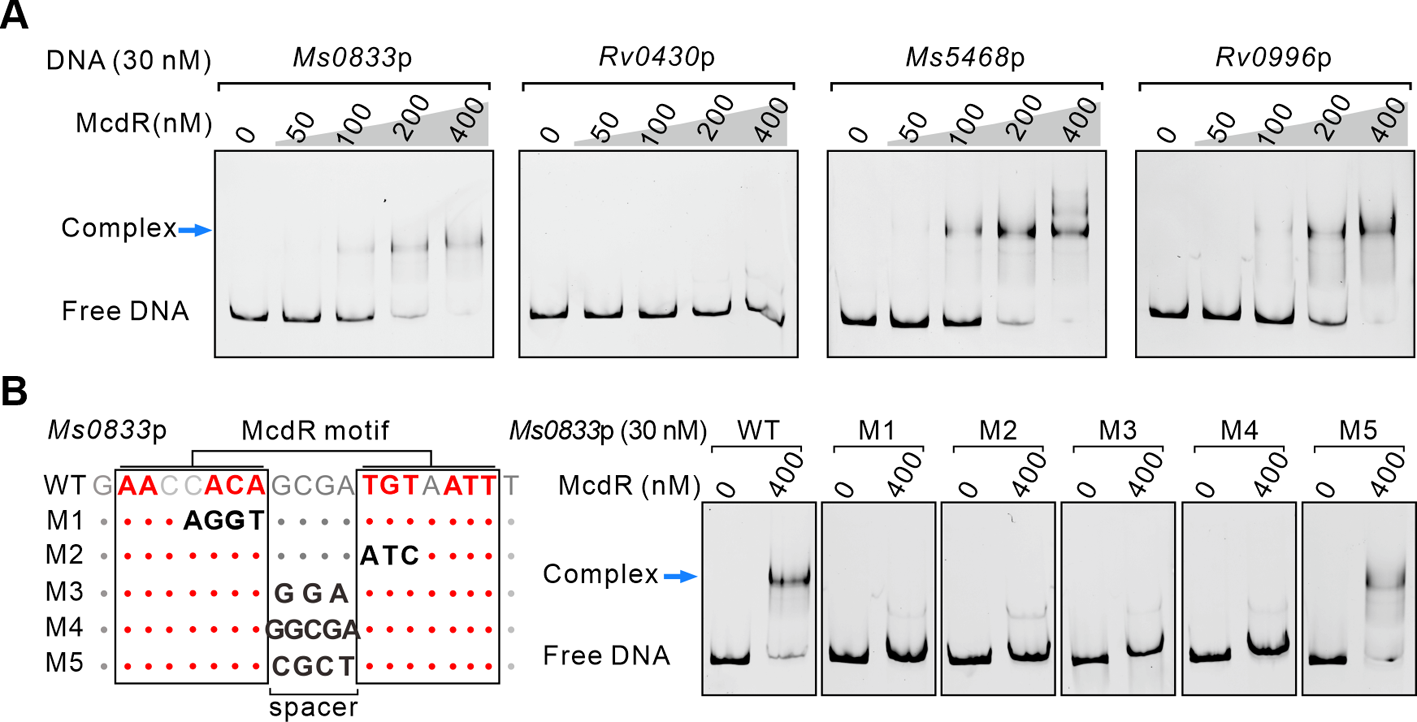

Supplement: FIG S4 [file mbio.03343-21-sf004.tif]

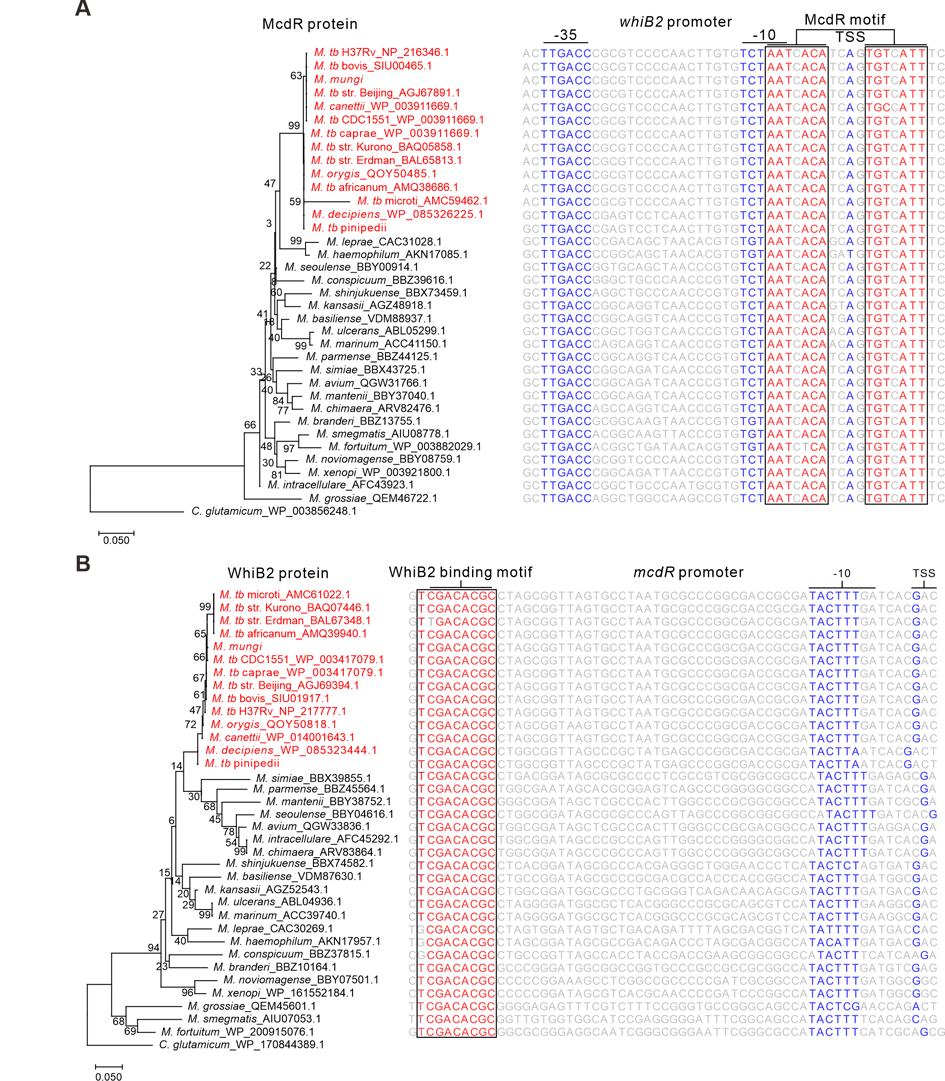

Supplement: FIG S5 [file mbio.03343-21-sf005.tif]

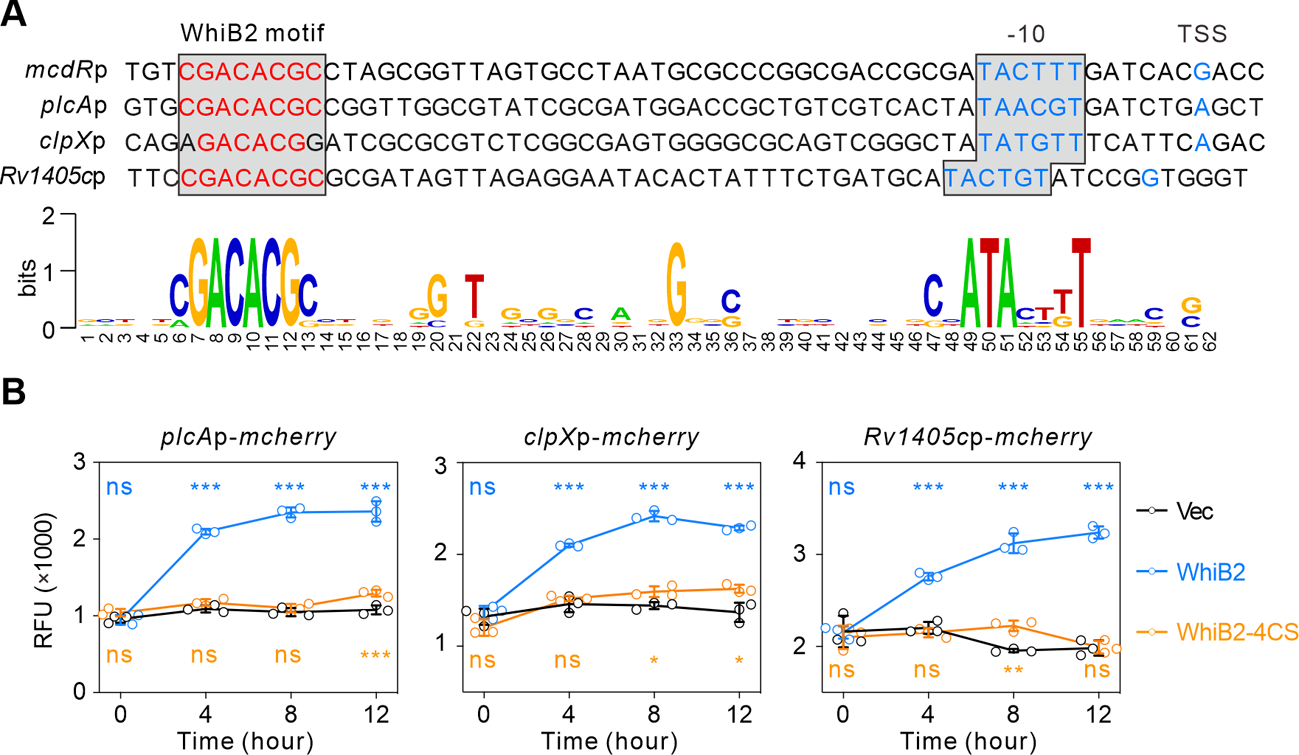

Supplement: FIG S6 [file mbio.03343-21-sf006.tif]
